# Human d‐lactate dehydrogenase deficiency by *LDHD* mutation in a patient with neurological manifestations and mitochondrial complex IV deficiency

**DOI:** 10.1002/jmd2.12220

**Published:** 2021-05-21

**Authors:** Anna Ka‐Yee Kwong, Sheila Suet‐Na Wong, Richard J. T. Rodenburg, Jan Smeitink, Godfrey Chi Fung Chan, Cheuk‐Wing Fung

**Affiliations:** ^1^ Department of Paediatrics and Adolescent Medicine, Li Ka Shing Faculty of Medicine The University of Hong Kong Hong Kong SAR China; ^2^ Department of Paediatrics and Adolescent Medicine Hong Kong Children's Hospital Hong Kong SAR China; ^3^ Radboud Centre for Mitochondrial Medicine, Department of Paediatrics Radboud Institute for Molecular Life Sciences, Radboud University Nijmegen Medical Centre Nijmegen The Netherlands

**Keywords:** ataxia, complex IV deficiency, d‐lactate dehydrogenase, developmental delay, *LDHD*, neurological

## Abstract

**Background:**

d‐lactate, one of the isomers of lactate, exists in a low concentration in healthy individuals and it can be oxidized to pyruvate catalyzed by d‐lactate dehydrogenase. Excessive amount of d‐lactate causes d‐lactate acidosis associated with neurological manifestations.

**Methods and Results:**

We report here a patient with developmental delay, cerebellar ataxia, and transient hepatomegaly. Enzyme analysis in the patient's skin fibroblast showed decreased mitochondrial complex IV activity. Using whole exome sequencing, we identified compound heterozygous variants in the *LDHD* gene, which encodes the d‐lactate dehydrogenase, consisting of a splice site variant c.469+1dupG and a missense variant c.752C>T, p.(Thr251Met) which are pathogenic and likely pathogenic respectively according to the American College of Medical Genetics and Genomics (ACMG) classification. The serum d‐lactate level was subsequently detected to be elevated (0.61 mmol/L, reference value: 0‐0.25 mmol/L).

**Conclusion:**

This is the third report on *LDHD* mutations associated with d‐lactate elevation and was first reported to have decreased mitochondrial complex IV activity. The study provides more information on this rare metabolic condition but the association of *LDHD* deficiency with the clinical presentations requires further investigations.

## 1 INTRODUCTION

l‐lactate and d‐lactate exist as two different optical isomers in the human body. l‐lactate, produced from anaerobic glycolysis, has concentration 100 times higher than d‐lactate in circulation.^1^ Circulating d‐lactate exists in a very low concentration (5‐20 μmol/L) in normal individuals.^2^ It is derived from exogenous source including food consumption, intestinal carbohydrate‐fermenting bacteria production, or endogenously in a minor methylglyoxal metabolism pathway converting toxic methylglyoxal to d‐lactate.^3^ d‐lactate can be converted to d‐pyruvate catalyzed by d‐lactate dehydrogenase which has been identified and well‐characterized in lower organisms such as bacteria and yeasts.^4^, ^5^, ^6^ Mammalian d‐lactate dehydrogenase was later identified with high similarity to yeast d‐lactate dehydrogenase 1 with primary intracellular location in mitochondria.^7^ A recent study demonstrated that human wild‐type *LDHD* encoding human d‐lactate dehydrogenase, but not mutated *LDHD* from a patient, could rescue the phenotype of d‐lactate metabolism by *LDHD* knockout zebrafish model and it provides evidence that *LDHD* is responsible for human d‐lactate metabolism.^1^

Lactic acidosis is a medical condition which is resulted from the accumulation of excess lactate, usually l‐lactate, and proton in body fluids with poor clinical outcomes.^8^ Comparing with l‐lactate acidosis, d‐lactate acidosis is a relatively rare condition which is also known as d‐lactate encephalopathy.^9^ Both l‐lactate and d‐lactate can pass the blood‐brain barrier. Unlike its levorotary counterpart, elevation of d‐lactate has neurotoxic effects resulting in neurological symptoms.^9^, ^10^ The presenting neurological signs of d‐lactate encephalopathy include slurred speech, ataxia, gait disturbance, altered mental condition, and behavioral change which would often be confused with primary neurological disorders.^9^

Recently, elevation of d‐lactate associated with neurological phenotypes has been reported in two patients with d‐lactate dehydrogenase deficiency resulting from *LDHD* mutations.^1^ So far, there are only two studies reporting *LDHD* mutations associated with d‐lactate elevation.^1^, ^11^ Here, we report another patient with *LDHD* mutations with increased serum d‐lactate level. The patient presented with global developmental delay, ataxia, transient hepatomegaly, and mitochondrial complex IV deficiency. This is the third study of *LDHD* mutation associated with high d‐lactate level and first identification of mitochondrial complex deficiency in this disorder.

## 2 CASE REPORT

### 2.1 Clinical history

Our patient was 14 years old and born of a nonconsanguineous Chinese couple with no relevant family history. She presented with global developmental delay since infancy. Her Griffiths Mental Developmental Scale at 32.5 months showed that her mental age was only 21 months. Mild hepatomegaly was detected since 28 months without an identifiable cause, which resolved after 3 months. Mild cerebellar ataxia was detected at 5 years of age with resolution after 10. Central hypotonia was mild all along. Her growth parameters including head circumference, height, and weight have been along the 50th to 75th percentile. The latest intellectual assessment by Wechsler Intelligence Scale for Children‐Fourth Edition (Hong Kong) (WISC‐IV) at 13 years old revealed a full‐scaled intelligence quotient of 76 which was in the limited intelligence range. Magnetic resonance imaging of the brain was normal. Liver function and ultrasonography was normal. Currently, the patient is not put on any dietary restrictions. She is studying in a mainstream school with special educational needs for her learning difficulty.

Metabolic workup showed intermittent hyperlactatemia with highest lactate 3.22 mmol/L (0.55‐2 mmol/L). The plasma amino acid profile was unremarkable with normal levels of branched chain amino acid. She had persistent increase in lactate and organic acids excretion including 3‐hydroxybuyrate, 2‐hydroxyisovalerate, 2‐hydroxy‐3‐methylvalerate, and 2‐hydroxyisocaproate in urine. Blood uric acid was marginally raised at 316 μmol/L (normal range: 105‐300 μmol/L).

### 2.2 Enzyme analysis

Measurements of pyruvate dehydrogenase (PDH) and respiratory chain complex activities in the patient's skin fibroblasts were performed at the Radboud Centre for Mitochondrial Medicine, The Netherlands. It revealed reduction in complex IV activity (221 mU/UCII; reference: 288‐954). The enzyme measurements were repeated in a new fibroblast culture and confirmed the mild complex IV deficiency (259 mU/U CS; reference: 288‐954). Enzyme activities were repeatedly within normal ranges for the other oxidative phosphorylation complexes including complex I, complex II, complex III, succinate: cytochrome c oxidoreductase, and complex V. Although a PDH‐E3 deficiency was suspected because of the hyperlactatemia and raised branched chained amino acid metabolites in the urine, both the activity of the PDH holoenzyme and of PDH‐E3 were within normal ranges.

### 2.3 Genetic analysis

**FIGURE 1:**
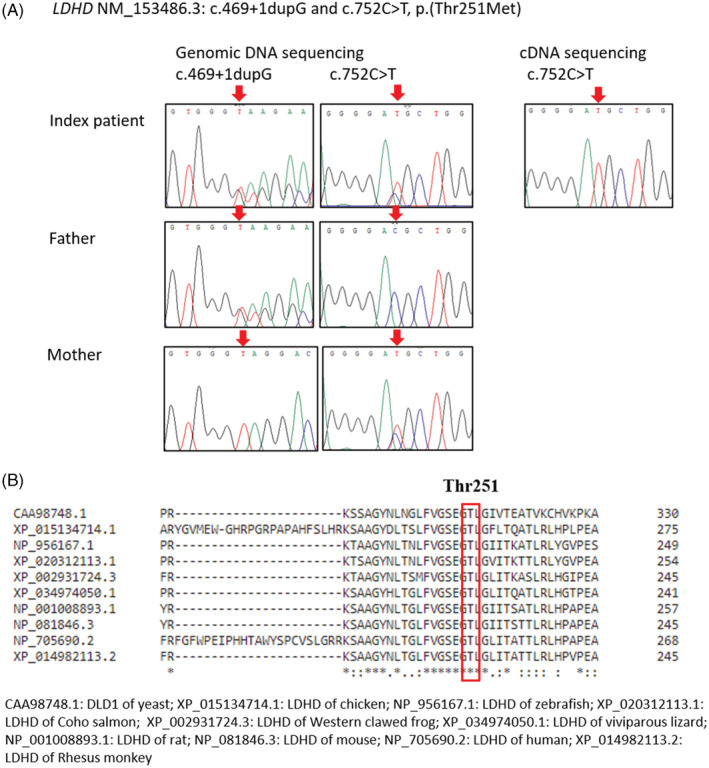
Identification of human *LDHD* variants. **A**, Sanger sequencing of index patient, father and mother. Two *LDHD* heterozygous variants, NM_153486.3: c.469+1dupG and c.752C>T, p.(Thr251Met) were confirmed by genomic DNA sequencing. Sanger sequencing of the cDNA revealed that only one allele with the missense variant was expressed. Father is heterozygous carrier of the splice site variant c.469+1dupG and the mother is heterozygous carrier of the missense variant p.(Thr251Met). Arrows point to the specific nucleotide changes. B, Multiple alignments of LDHD amino acid sequences across yeast and different vertebrates showed that the variant p.(Thr251Met) encodes for the amino acid methionine (Met) instead of the normally present amino acid threonine (Thr) in a region that is highly conserved. cDNA, complementary DNA

Whole exome sequencing (WES) and bioinformatics analyses were performed as described previously.^12^, ^13^ The variants called were annotated by Oncotator version 1.8.0.0 and filtered by first‐tier variant analysis based on a virtual gene panel associated with mitochondrial diseases and with strong support of mitochondrial localization suggested in MitoCarta 2.0. Compound heterozygous variants were identified in the *LDHD* gene. These variants have been reported in our previous study on a patient cohort suspicious of mitochondrial diseases.^14^ The variants were further confirmed by Sanger sequencing. One of the variants was intronic within the splice site (c.469+1dupG, NM_153486.3) (Figure 1A) which was predicted to alternate wild‐type donor site and most probably affect splicing by online software Human Splicing Finder.^15^ Another variant was a missense one [c.752C>T, p.(Thr251Met), NM_153486.3] (Figure 1A). Both variants were not found in East Asian population according to The Genome Aggregation Database (gnomAD). Sanger sequencing of the identified variants in parental DNA was performed and showed that the variants were segregated between the parents. The father is the carrier of the intronic splice site variant and the mother is the carrier of the missense variant (Figure 1A).

In silico analysis including sorting intolerant from tolerant, Polyphen‐2 and Mutation Taster also predicted that this residue was located in highly conserved region and p.(Thr251Met) was predicted to be damaging to the protein structure and function. Multiple alignments of LDHD of human, eight vertebrates and DLD1 of yeast were performed by online Clustal Omega (1.2.4) multiple sequence alignment software,^16^ the amino acid residue of Thr251 was highly conserved evolutionarily (Figure 1B). Using the three‐dimensional modeling by structure‐based prediction of protein stability changes upon single‐point mutation, the p.(Thr251Met) variant results a delta‐delta G value of 2.0. A positive delta‐delta G value implies the variant is responsible for LDHD protein fold stabilization.^17^ According to ACMG classification,^18^ the frameshift variant falls into the tier of “Pathogenic.” It was suggested that nonsense mediated decay (NMD) degrades alternatively spliced (AS) transcript with reading frameshift and premature translation‐termination codon by a strategy called AS coupled to NMD (AS‐NMD).^19^ The missense variant falls into the tier of “Likely pathogenic” with a Post_P value 0.9 using the Bayesian classification framework.^20^

### 2.4 Reverse transcription polymerase chain reaction

RNA was extracted from the patient's fibroblasts and semiquantitative reverse transcription polymerase chain reaction was performed to amplify the *LDHD* complementary DNA (cDNA) consisting of the region with the two variants found. Sanger sequencing of the cDNA revealed that only the allele with the missense variant was expressed while cDNA with the splice site variant was not identified (Figure 1A). This finding illustrated that the mRNA with the splice site variant could be eliminated by AS‐NMD which targets transcripts with the aberrant splicing.

### 2.5 Quantitation of serum d‐lactate level

After identification of *LDHD* mutations, the patient's serum d‐lactate level was quantified in Mayo Clinic Laboratories. d‐lactate is oxidized to pyruvate in the presence of d‐lactate dehydrogenase and nicotinamide adenine dinucleotide (NAD). The quantity of reduced NAD produced, which was directly proportional to the level of d‐lactate oxidized, was measured spectrophotometrically at 340 nm.^21^, ^22^ Serum d‐lactate level was found to be higher than normal (0.61 mmol/L; reference value: 0‐0.25 mmol/L).

## 3 DISCUSSION

### 3.1 Comparisons of the previous two studies of LDHD deficiency

This is the third study reporting *LDHD* mutations associated with d‐lactate elevation. Comparisons of the clinical presentation, metabolic profiles and genetic analyses of the three studies are summarized in Table 1.

**TABLE 1:** Comparisons of clinical features, metabolic profiles, and genetic analysis of the three studies of *LDHD* deficiency

	Monroe et al.^1^	Monroe et al.^1^	Drabkin et al.^11^	Our patient
Ancestry	Sicilian village in Italy	Moluccan in Indonesia	Bedouin‐Israeli	Chinese
Parents	Originated from the same Sicilian village share some degree of consanguinity.	Consanguineous	Consanguineous	Nonconsanguineous
Age of onset of symptoms and signs	1 y old	5 mo. old	Gout arthropathy has been reported in adult patients but ages of onset were not mentioned	Infancy
Age at the time of publication	40 y old	Not mentioned	The kindred included both adults and children, but ages were not mentioned.	14 y old
Neurological and developmental outcome	Delayed motor and mental development, intellectual disability, microcephaly, epilepsy, dysmorphic features, and behavioral problems	West syndrome, experienced developmental regression with severe hypotonia including head lag, lost social interaction and remains developmentally delayed	No neurological symptoms reported	Global developmental delay evolving into limited intelligence, transient ataxia, central hypotonia
Other clinical features	Bilateral inguinal hernia, cryptorchidism, mildly dysplastic helices and aniridia	Not mentioned	Gout arthropathy upper‐ and lower‐limb joint pain, particularly in small joints of the palms and toes, with acute gout flares every 3‐6 mo.	Transient hepatomegaly in infancy
d‐lactate	Plasma d‐lactate concentration: 0.7 mM	Plasma d‐lactate concentration: 1.1‐1.2 mM	Average plasma d‐lactate concentration: 3.16 ± 0.63 mM	Serum d‐lactate concentration: 0.61 mM
Organic acid	Elevation of 2‐hydroxyisovaleric acid and 2‐hydroxyisocaproic acid in plasma and urine	Elevation of 2‐hydroxyisovaleric acid and 2‐hydroxyisocaproic acid in plasma and urine	Not mentioned	Increase in excretion of 3‐hydroxybuyrate, 2‐hydroxisovalerate, 2‐hydroxy‐3‐methylvalerate, and 2‐hydroxyisocaproate in urine
Uric acid	Not mentioned	Not mentioned	Elevation of plasma uric acid levels; Average plasma uric acid levels were 10.34 ± 1.84 mg/dL (615 ± 109 μmol/L) and 6.75 ± 0.7 mg/dL (401 ± 42 umol/L) in the affected adults and children (normal levels are 208‐428 and 119‐369 μmol/L)	Elevation of plasma uric acid levels (316 μmol/L, normal range: 105‐300 μmol/L)
Respiratory chain enzyme activities	Not mentioned	Not mentioned	Not mentioned	Moderate reduction in complex IV activity
*LDHD* variants identified	Homozygous missense *LDHD* variant c.1388C>T, p.(Thr463Met) (NM_153486.3)	Homozygous missense *LDHD* variant c.1122G>T, p.(Trp374Cys) (NM_153486.3)	Homozygous missense LDHD variant c.1108C>T, p.(Arg370Trp) (NM_153486.3)	Compound heterozygous splice site variant c.469+1dupG, NM_153486.3) and missense variant c.752C>T, p.(Thr251Met) (NM_153486.3)
Method for variants identification	Analysis of homozygosity regions by single nucleotide polymorphism (SNP) array for genes related to d‐lactate excretion	Sanger sequencing after identification of the first LDHD variant in the same study	Linkage analysis and homozygosity mapping. Whole exome sequencing and filtration of homozygous variants	Whole exome sequencing with first tier analysis of genes associated with mitochondrial diseases and with strong support of mitochondrial localization suggested in MitoCarta 2.0
Other genotypes possibly explaining the clinical features	Array comparative genomic hybridization analysis revealed a de novo 11p13 deletion explaining the neurodevelopmental features	Trio whole exome sequencing identified de novo variant in CACNA1B (NM_000718.3: c.1429C>T, p.(Arg477Cys). This gene was not previously been linked to West Syndrome and may be candidate for epilepsy phenotypes.	No chromosomal/genomic changes by array comparative genomic hybridization analysis and no other genotype were identified to explain the clinical features.	Whole exome sequencing did not identify any pathogenic variants associated with neurodevelopmental features.

The *LDHD* mutations found in the first and second study were homozygous *LDHD* variants because the affected individuals are from consanguineous marriages. These homozygous variants were identified by analysis of homozygosity regions by SNP array in the first study^1^ and WES followed by filtration of homozygous variants in the second study.^11^ Compound heterozygous *LDHD* variants were identified in our patient through WES, demonstrating that WES is a powerful molecular diagnostic tool for discovery of uncommon genetic defect in unresolved rare diseases while the clinical signs are not specific enough for targeted gene analysis.

The clinical presentation and metabolic profiles of our patient were comparable to the two patients of the first study.^1^ Both patient 1 and our patient showed delayed development. Both patient 2 and our patient presented with central hypotonia. All three patients showed increased circulating d‐lactate level and elevation of specific organic acids in urine. The elevation of d‐lactate level could be explained by the mutation of *LDHD* encoding for human d‐lactate dehydrogenase and the study of Monroe et al^1^ provided evidence that *LDHD* is responsible for human d‐lactate metabolism. The specific pattern of urine organic acid in both studies also suggested a role of *LDHD* in the metabolism of branched‐chained ketoacids but further studies are necessary to clarify the metabolic pathway(s) involved.

Clinical presentation of the patient in the second report^11^ is quite different from the patients in both the first report and in our study. The affected members of the kindred presented with hyperuricemia and gout arthropathy with no neurological symptoms reported. The circulating d‐lactate levels are higher than those reported in the first study and in our study. This high d‐lactate level is comparable to the level reported in d‐lactate acidosis in short bowel syndrome but no neurological manifestation was reported. Organic acid level of the kindred has not been measured. Blood uric acid of our patient was marginally raised at 316 μmol/L (normal 105‐300 μmol/L) and no gout arthropathy was identified. A possible explanation is that our patient is still young and from the second paper, they only reported that all affected adults were clinically diagnosed with gout arthropathy and the average uric acid level in children was also marginal (401 ± 42 μmol/L; normal levels 119‐369 μmol/L). Active follow‐up on the uric acid level with any joint involvement will be essential for our patient. For the relationship between d‐lactate and uric acid elevation, previous study suggested that lactate and organic acid including beta‐hydroxybutyrate may compete with and reduce the renal clearance of uric acid leading to high level of serum uric acid associated with gout.^23^, ^24^, ^25^

The clinical features in different reports are variable. The kindred in the second report with the highest level of d‐lactate seems to have the mildest presentation, we cannot exclude the possibility that the phenotypes are resulted from other genetic defects rather than d‐lactate dehydrogenase deficiency as they have consanguineous background. Besides, the neurological signs of the two patients in the first study could be potentially explained by the additional genetic defects identified. Despite this, we cannot exclude the possible association of *LDHD* deficiency with neurological phenotypes as d‐lactic acidosis has potential neurotoxic effects leading to neurological symptoms. Other than the clinical presentations, the metabolic phenotypes can be well explained by *LDHD* mutations in all the reports. More evidence and case studies are required to support the relationship of *LDHD* deficiency and clinical manifestations. Currently, transcriptomic analysis is ongoing in our patient to further explore the underlying patho‐mechanism of *LDHD* deficiency and the possibility of mutation(s) involving a second gene which could have explained the clinical phenotype.

### 3.2 Ataxia, transient hepatomegaly, and decrease in mitochondrial complex IV activity

In addition to developmental delay and hypotonia, our patient presented with ataxia which were not reported in the previous cases. Ataxia is one of the common neurological manifestations presented during d‐lactate acidosis in patients with short bowel syndrome and other d‐lactate encephalopathy..^9^, ^26^ Among these neurological symptoms, ataxia was third common symptom of d‐lactic acidosis in 32% of patients with short bowel syndrome as reviewed previously.^9^

Our patient also has transient mild hepatomegaly in infancy without major precipitating causes such as infection. From GTEx expression data (https://www.gtexportal.org/home) and result from the second study using reverse transcription‐polymerase chain reaction,^11^ human *LDHD* transcripts were abundant in tissues with a high metabolic rate in which liver, skeletal muscle, heart, and colon had the highest expression levels. d‐lactate is produced in the methylglyoxal pathway in the liver cytosol.^27^ A previous study demonstrated that rat liver mitochondria can take up external d‐lactate, metabolize it by d‐lactate dehydrogenase located in mitochondrial inner membrane.^28^ In this way, d‐lactate dehydrogenase is essential for the removal of methylglyoxal which is toxic to the liver and *LDHD* deficiency in our patient may be the cause of hepatomegaly in infancy. However, her liver function was all along normal. The reason for the transient nature of liver involvement remained elusive.

An interesting finding is the reduction of mitochondrial complex IV activity in our patient. Prior to the enzyme measurements, the cells were cultured in cell culture medium for several passages during which the medium was refreshed several times. Therefore, it seems unlikely that elevated d‐lactate as observed in the patient's plasma is responsible for the reduced complex IV activity observed in the cultured fibroblasts. The association of d‐lactate dehydrogenase with mitochondrial respiratory chain has been studied in lower organisms but not well known in mammals. Previous study in plant suggested that d‐lactate dehydrogenase delivered electrons from methylglyoxal degradation to the respiratory chain through cytochrome C.^29^ In bacteria, d‐lactate dehydrogenase contains a flavin adenine dinucleotide (FAD) binding site for FAD cofactor transferring electrons from the substrate oxidation to the electron transport chain for ATP generation.^30^ In *Saccharomyces cerevisiae*, mitochondrial LDHD has been shown to be involved in methylglyoxal detoxification and to connect d‐lactate oxidation to the mitochondrial electron transport chain.^6^ Mammalian LDHD was identified with high similarity to yeast DLD1, including a potential FAD binding site.^7^ However, whether the enzyme is expressed in fibroblasts is at present unknown. The mechanism behind the interaction of the impaired d‐lactate oxidation pathway with the mitochondrial respiratory chain in LDHD deficient patients deserves further investigations.

## 4 CONCLUSION

In conclusion, the present study is the third report on *LDHD* deficiency associated with d‐lactate elevation. The clinical features including ataxia, transient hepatomegaly, and mitochondrial complex IV deficiency have not been reported previously. The association of *LDHD* deficiency with the neurological features and the additional clinical presentations remained elusive. More case studies on this metabolic condition are necessary to provide further information on the potential phenotypic spectrum.

Although *LDHD* deficiency is a rare disease, it is still worth considering for plasma d‐lactate measurement and urine organic acid analysis in patients with unexplained neurological symptoms such as developmental delay, ataxia, and epilepsy, as part of routine neurometabolic workup.

## CONFLICT OF INTEREST

J. S. is the CEO of Khondrion, a pharmaceutical company developing compounds to potentially treat mitochondrial disease. All the other authors declare that they have no conflict of interest.

## AUTHOR CONTRIBUTIONS

Conception and design of study: Anna Ka‐Yee Kwong, Cheuk Wing Fung; Drafting the manuscript: Anna Ka‐Yee Kwong, Cheuk Wing Fung, Sheila Suet‐Na Wong; Evaluation of manuscript for content: Anna Ka‐Yee Kwong, Cheuk Wing Fung, Sheila Suet‐Na Wong, Jan Smeitink, Godfrey Chi Fung Chan; Data analysis and interpretation: Anna Ka‐Yee Kwong, Cheuk Wing Fung, Richard J. T. Rodenburg.

## ETHICS STATEMENT

Ethical approval had been obtained from the Institutional Review Board (IRB) of the University of Hong Kong‐Hong Kong West Cluster (IRB Ref. No.: UW 11‐190). Written consent was obtained from the parents of the patient.
